# Network Pharmacology Combined With Clinical Retrospective Analysis to Investigate the Potential Mechanisms and Clinical Significance of Folate in Treating Spinal Cord Injury

**DOI:** 10.1002/prp2.70233

**Published:** 2026-03-12

**Authors:** Xiaolei Chu, Jiajia Liang, Jiaojiao Sun, Wenjie Liu, Lei Zhang, Zheng Xing, Qingwen Li, Qi Li

**Affiliations:** ^1^ Department of Rehabilitation Tianjin University Tianjin Hospital Tianjin China; ^2^ Tianjin Key Laboratory of Exercise Physiology and Sports Medicine Institute of Sport, Exercise and Health, Tianjin University of Sport Tianjin China; ^3^ Medical School Tianjin University Tianjin China

**Keywords:** clinical retrospective analysis, folate, molecular docking, network pharmacology, spinal cord injury

## Abstract

Folate is an indispensable nutrient involved in key biological processes, including enzymatic reactions, DNA replication, metabolic regulation, and methylation. Studies indicate that folate promotes neuronal regeneration and repair in patients with spinal cord injury (SCI); however, its precise mechanisms remain unclear. We employed network pharmacology to identify potential targets of folate for SCI treatment; we conducted a clinical retrospective study, selecting 50 SCI patients and 50 non‐SCI control subjects from Tianjin Hospital between 2022 and 2025 to validate predictions by assessing serum folate levels. Network pharmacology identified 1402 folate‐related targets and 548 SCI‐related targets. Key targets included TNF‐α, CASP3, EGF, IL1β, and AKT1. Molecular docking revealed the highest binding affinity between folate and CASP3/TNF‐α (−8.5 kcal/mol). Clinical validation demonstrated statistically significant lower folate levels in SCI patients compared to normal levels in non‐SCI controls. A strong inverse correlation was observed between folate levels and injury level (*r* = −0.58, *p* < 0.001). Folate exerts neuroprotective effects by synergistically regulating neuronal apoptosis, inflammatory responses, and oxidative stress pathways. Clinical data reveal prevalent folate deficiency in SCI patients, and this nutritional deficit may exacerbate secondary injury cascades. We recommend incorporating folate supplementation into comprehensive SCI management protocols.

**Abbreviations**
BSCB: blood‐spinal cord barrier
GO: gene ontology
KEGG: Kyoto Encyclopedia of Genes and Genomes
PPI: protein–protein interaction
SCI: spinal cord injury

## 1 Introduction

Spinal cord injury (SCI) is a central nervous system disorder characterized by structural damage resulting from trauma, disease, or degenerative changes, typically causing sensory, motor, and autonomic dysfunction below the injury level [1]. According to the World Health Organization, approximately 250 000 to 500 000 new SCI cases occur annually worldwide [2], with incidence rates from traffic accidents, falls, and occupational injuries showing a progressive increase [3]. Patients with SCI exhibit poor prognoses requiring long‐term rehabilitation and specialized care. In the United States, annual expenditures for SCI treatment and disability management exceed $14 billion [4], imposing substantial socioeconomic burdens on individuals, families, and society. The pathophysiology of SCI involves primary and secondary injury phases. Following initial mechanical damage, impaired spinal cord circulation triggers neuronal and glial cell death, local tissue ischemia, and inflammation, ultimately culminating in secondary injury [5]. Critically, inflammation‐driven microenvironmental dysregulation represents a key therapeutic target for neural repair [6]. Previous studies demonstrate that suppressing neuroinflammation reduces inflammatory mediator expression, mitigates secondary damage, and creates a permissive microenvironment for axonal regrowth [7]. Similarly, improving local ischemic–hypoxic conditions attenuates secondary injury and enhances neurological recovery [8]. Current clinical management of acute SCI relies primarily on surgical intervention combined with high‐dose methylprednisolone (MP). Although this approach may transiently improve outcomes, its significant adverse effects—including elevated risks of wound infections, gastrointestinal hemorrhage, sepsis, pulmonary embolism, and mortality—cannot be overlooked [9]. Consequently, developing novel therapeutic strategies that effectively modulate inflammation, ameliorate ischemic–hypoxic microenvironments, and promote neural repair is imperative.

Folate (vitamin B9) is an essential cofactor in enzymatic reactions involving DNA replication, nucleotide synthesis, amino acid metabolism, and methylation processes [10]. Clinically, it prevents neural tube defects and restores hematopoietic function in folate‐deficiency macrocytic anemia [11]. As the essential methyl donor in eukaryotes, folate metabolism provides functional methyl groups for epigenetic regulation of DNA, RNA, and histones. This epigenetic regulatory mechanism exhibits cross‐tissue conservation, promoting regenerative processes not only in the central nervous system (CNS) but also in liver, skin, and bone [12]. Folate critically maintains neuronal and glial membrane integrity through lipid protection [13]. Notably, folate administration enhances regeneration of injured spinal axons through DNA methylation‐dependent mechanisms. Intraperitoneal folate treatment in adult rats significantly improves sensory axon regeneration into peripheral nerve grafts [14]. Furthermore, folic acid supplementation enhanced neurological recovery after spinal cord contusion, with therapeutic effects extending beyond embryonic development to effectively support growth, repair, and functional recovery in the adult CNS [15]. Despite evidence supporting folate's therapeutic potential for SCI, critical knowledge gaps persist regarding its systematic mechanisms and clinical efficacy. Network pharmacology—integrating bioinformatics and pharmacological methods—provides a powerful framework for visualizing drug‐target‐disease interactions and guiding therapeutic development [16]. In this study, we employed network pharmacology combined with clinical retrospective analysis to identify folate's therapeutic targets and mechanisms in SCI repair.

## 2 Results

### 2.1 Active Ingredients and Targets of Folate

**FIGURE 1:**
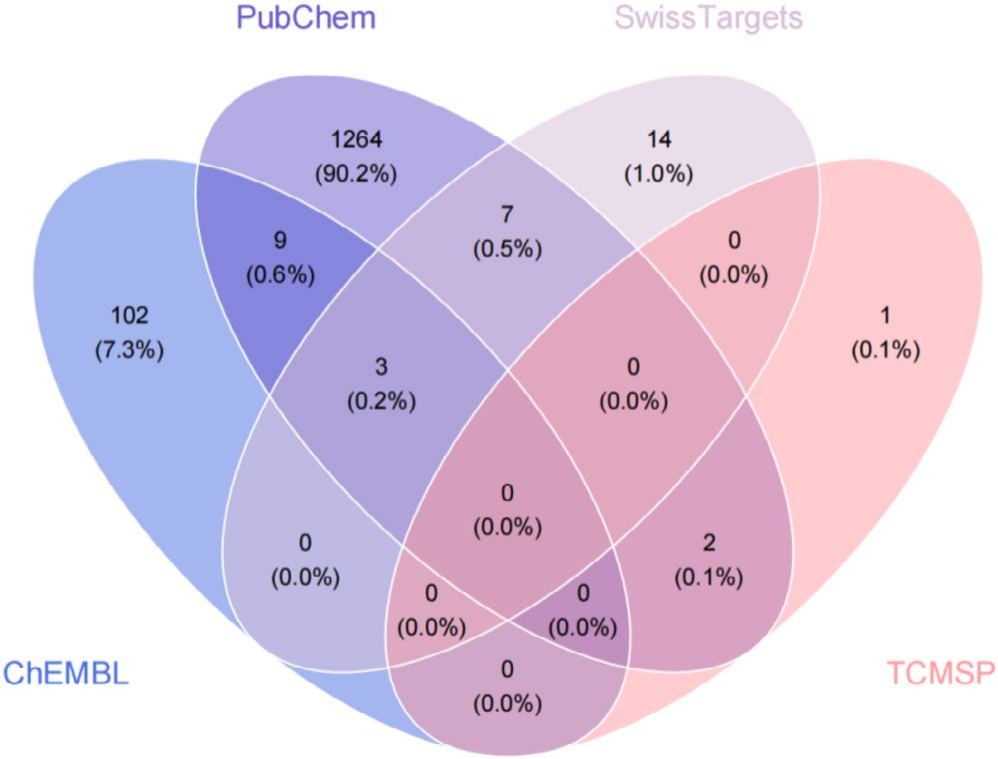
Folate‐related targets.

Active ingredients were retrieved from the TCMSP database by screening for compounds meeting the criteria of oral bioavailability ≥ 30% and drug‐likeness ≥ 0.18. Additional active ingredients and their targets were identified using the SwissTargetPrediction, ChEMBL, and PubChem databases (Figure 1 shows the number of folate‐related targets). After removing duplicates, a total of 1402 unique targets were obtained.

### 2.2 Predicted SCI‐Related Targets

**FIGURE 2:**
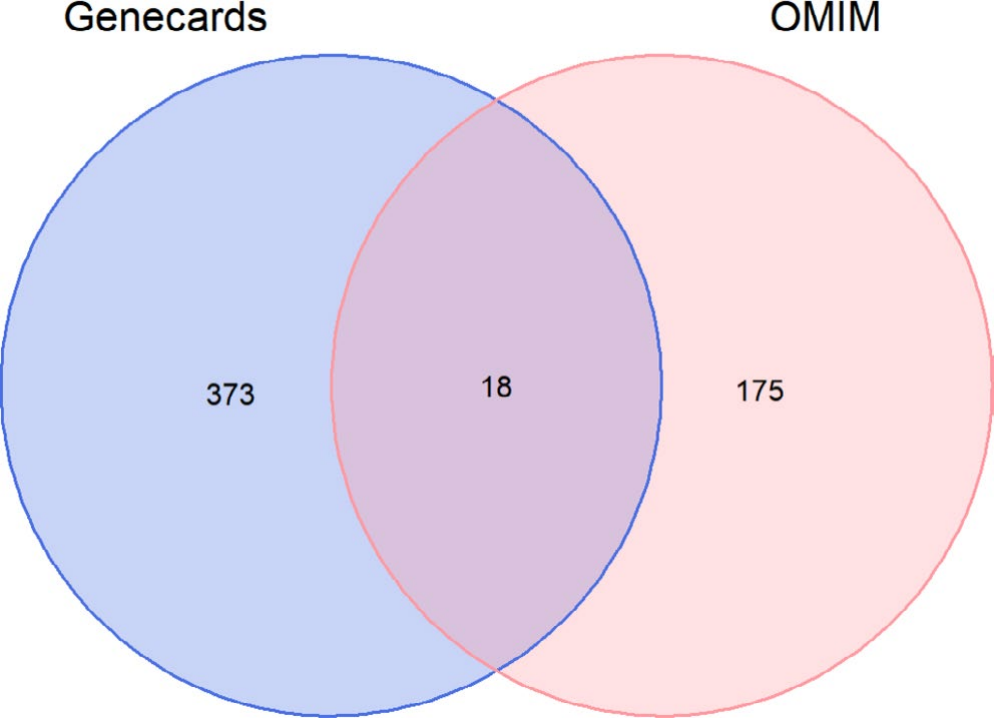
SCI‐related targets.

Using the keyword “Spinal cord injury” and a relevance score > 1 as the screening criterion, 391 and 193 disease‐associated targets were retrieved from the GeneCards and OMIM databases, respectively. These targets were integrated and deduplicated, yielding 548 unique SCI‐related targets (Figure 2).

### 2.3 Construction of the Folate Component‐Target Network for SCI

**FIGURE 3:**
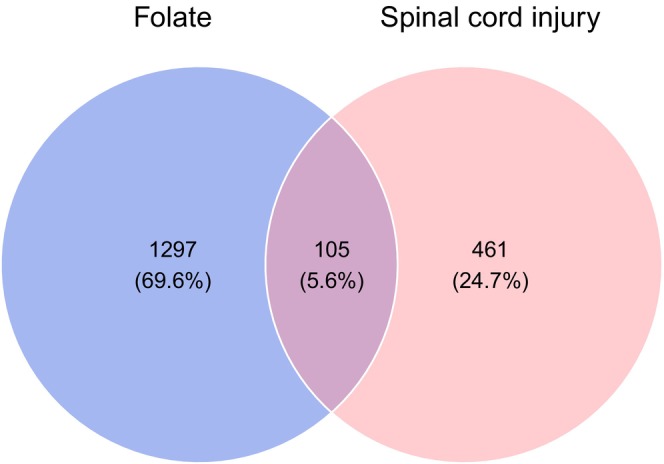
Target prediction of folate for SCI.

**FIGURE 4:**
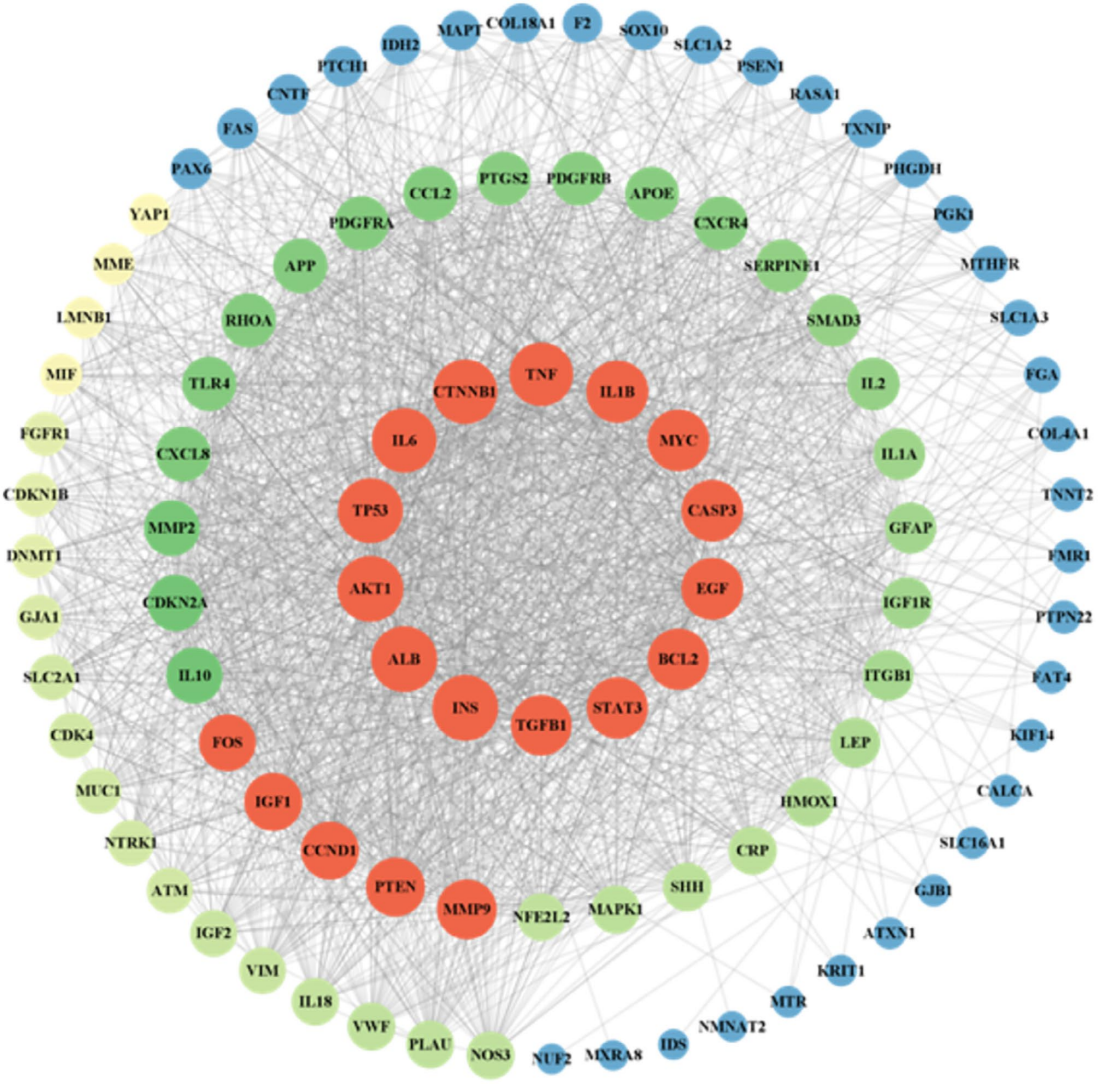
Drug‐Ingredient‐Target network construction.

The intersection between folate targets and SCI‐related targets yielded 105 potential therapeutic targets. A Venn diagram was generated using the Xiantao Academic Bioinformatics Platform (Figure 3), illustrating the number of common targets between SCI and folate. The “Drug‐Ingredient‐Target” network was constructed (Figure 4).

### 2.4 PPI Network

**FIGURE 5:**
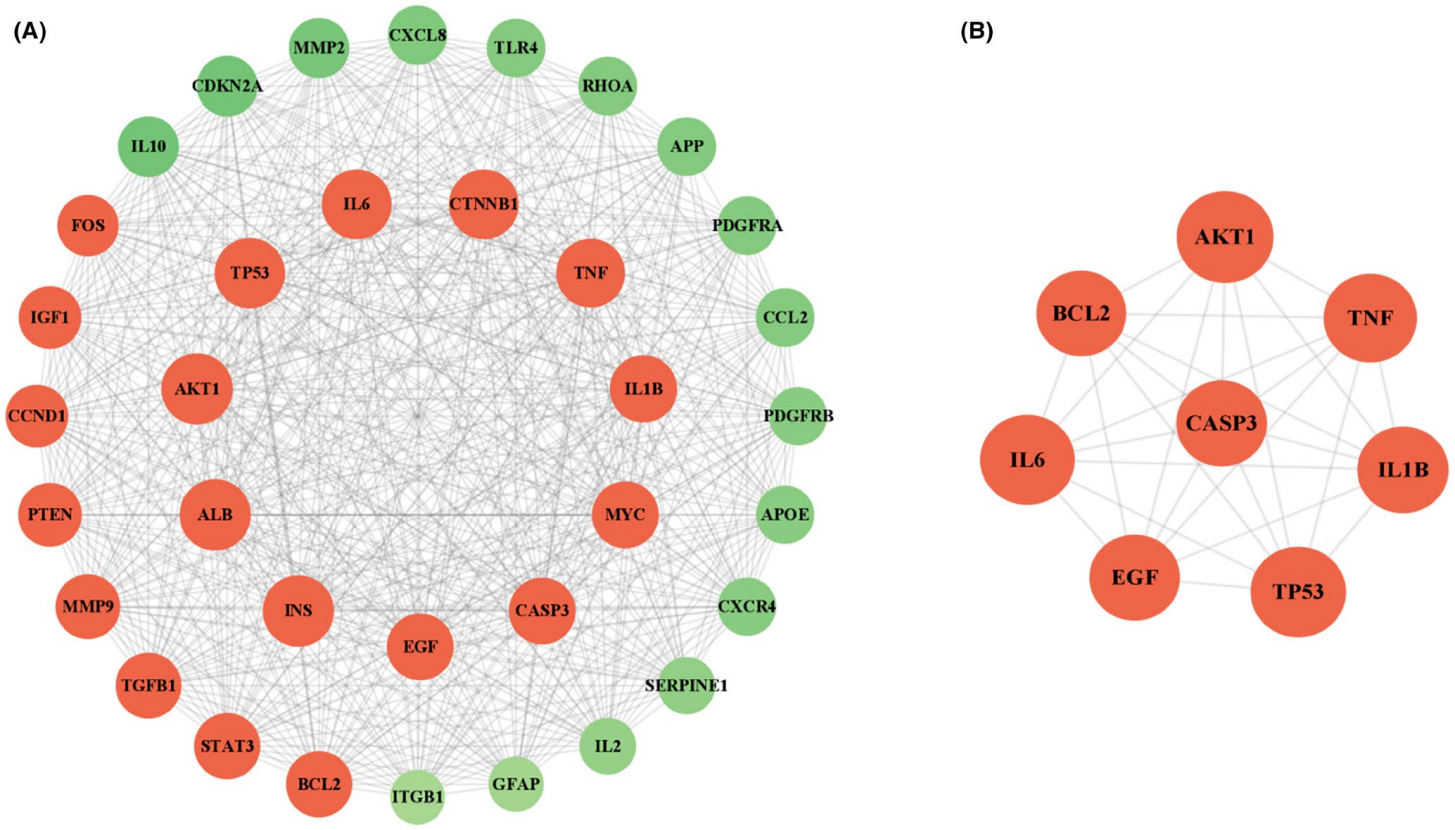
PPI Results; (A) Protein–protein interactions among potential targets; (B) Core target interaction network extracted using CYTOSCAPE 3.9.1.

Potential therapeutic targets of folate for SCI were imported into the STRING platform to construct the PPI network (Figure 5). Within the PPI network, degree, closeness centrality, and betweenness centrality represent the number of direct connections to a node, the reciprocal of the average shortest path length from the node to all other nodes, and the number of shortest paths passing through the node, respectively. Targets with degree > 10, closeness centrality > 0.4, and betweenness centrality > 0.03 were considered significant [17]. Using the CytoNCA plugin, 8 key proteins potentially targeted by folate in relation to SCI were identified: Tumor Necrosis Factor‐α (TNF‐α), Interleukin‐1 Beta (IL1β), TP53 regulating kinase (TP53), Interleukin‐6 (IL 6), RAC‐alpha serine/threonine‐protein kinase 1 (AKT1), Caspase‐3 (CASP3), Epidermal Growth Factor (EGF), and B‐cell lymphoma 2 (BCL2).

### 2.5 GO Term and KEGG Pathway Enrichment

**FIGURE 6:**
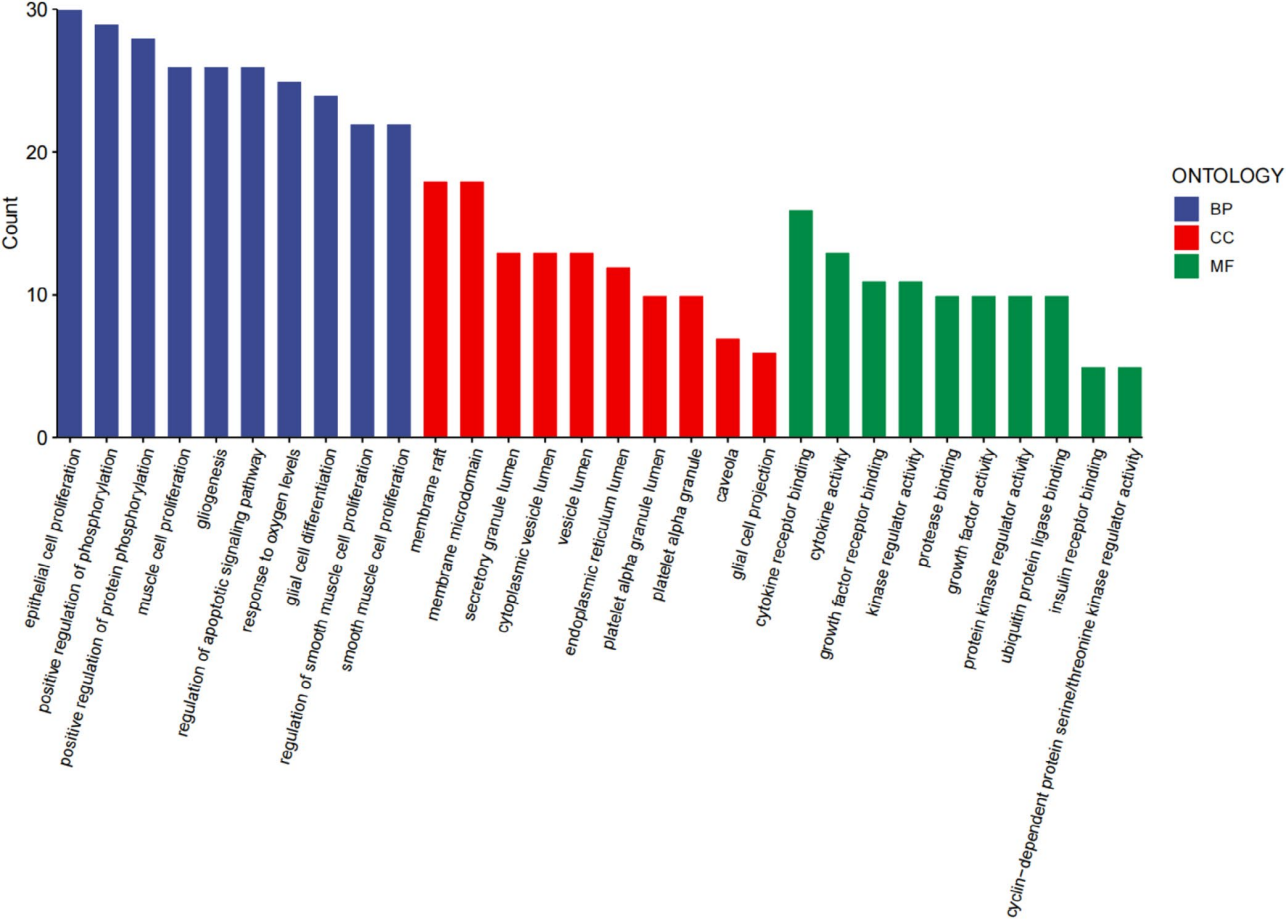
GO Functional Enrichment Analysis; Targets were categorized into three domains: Biological Processes (BPs), Cellular Components (CCs), and Molecular Functions (MFs). This analysis provides insights into biological processes, cellular localization, and molecular activities associated with the targets. Higher enrichment scores indicate stronger relevance.

GO functional enrichment analysis of folate's potential targets for SCI revealed 2437 BPs, 298 CCs, and 89 MFs. The top 10 enriched terms for each category, selected based on *p*‐value and count, were visually analyzed (Figure 6). The top 10 enriched BPs were primarily associated with muscle cell proliferation, glial cell differentiation, and regulation of apoptotic signaling pathways, suggesting folate may promote SCI recovery by enhancing glial cell function, inhibiting apoptosis, and boosting neuronal proliferation. The top 10 enriched CCs included the synaptic membrane, vesicle lumen, and endoplasmic reticulum lumen, indicating folate might influence neurotransmitter synthesis, storage, and release, thereby improving synaptic function and neuronal signaling capacity. The top 10 enriched MFs involved growth factor receptor binding, protein kinase regulatory activity, and ubiquitin ligase activity, suggesting folate may impact neural repair and regeneration by modulating growth factor signaling and protein homeostasis.

**FIGURE 7:**
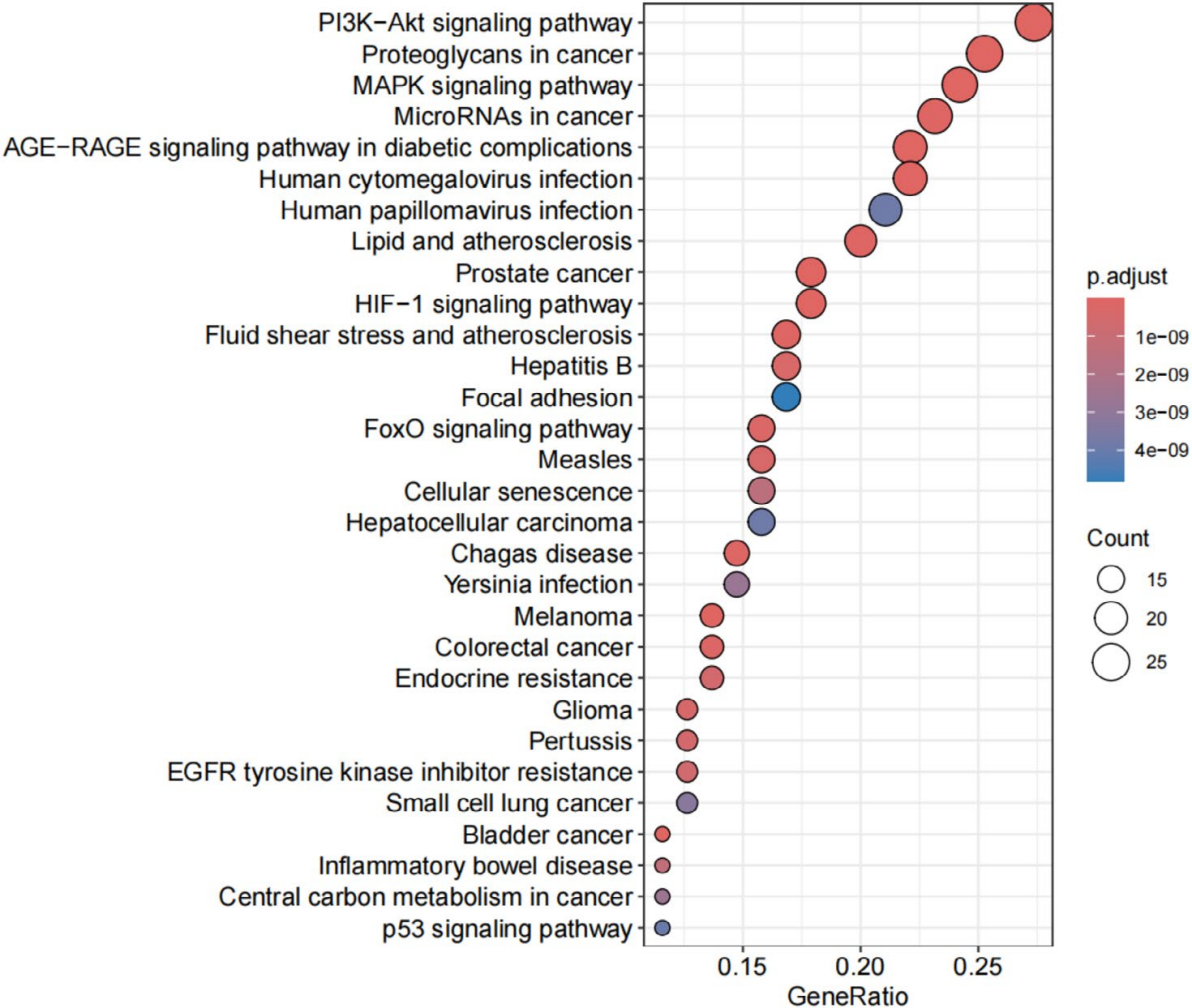
KEGG pathway enrichment analysis. Significantly enriched pathways were identified. Results are presented as a histogram where color gradients represent decreasing −log(*p*) values, and sphere size reflects enrichment level (larger spheres indicate greater enrichment strength).

KEGG pathway enrichment analysis identified 95 pathways, primarily associated with regulating cell survival, anti‐apoptosis, and neuroprotection (Figure 7). Notable pathways included the AGE‐RAGE signaling pathway, PI3K‐Akt signaling pathway, and MAPK signaling pathway. The results indicated that these genes are involved in biological pathways related to SCI repair and neural regeneration, suggesting folate may promote SCI recovery by modulating cell survival, anti‐apoptotic, and neuroprotective pathways.

### 2.6 Molecular Docking

**FIGURE 8:**
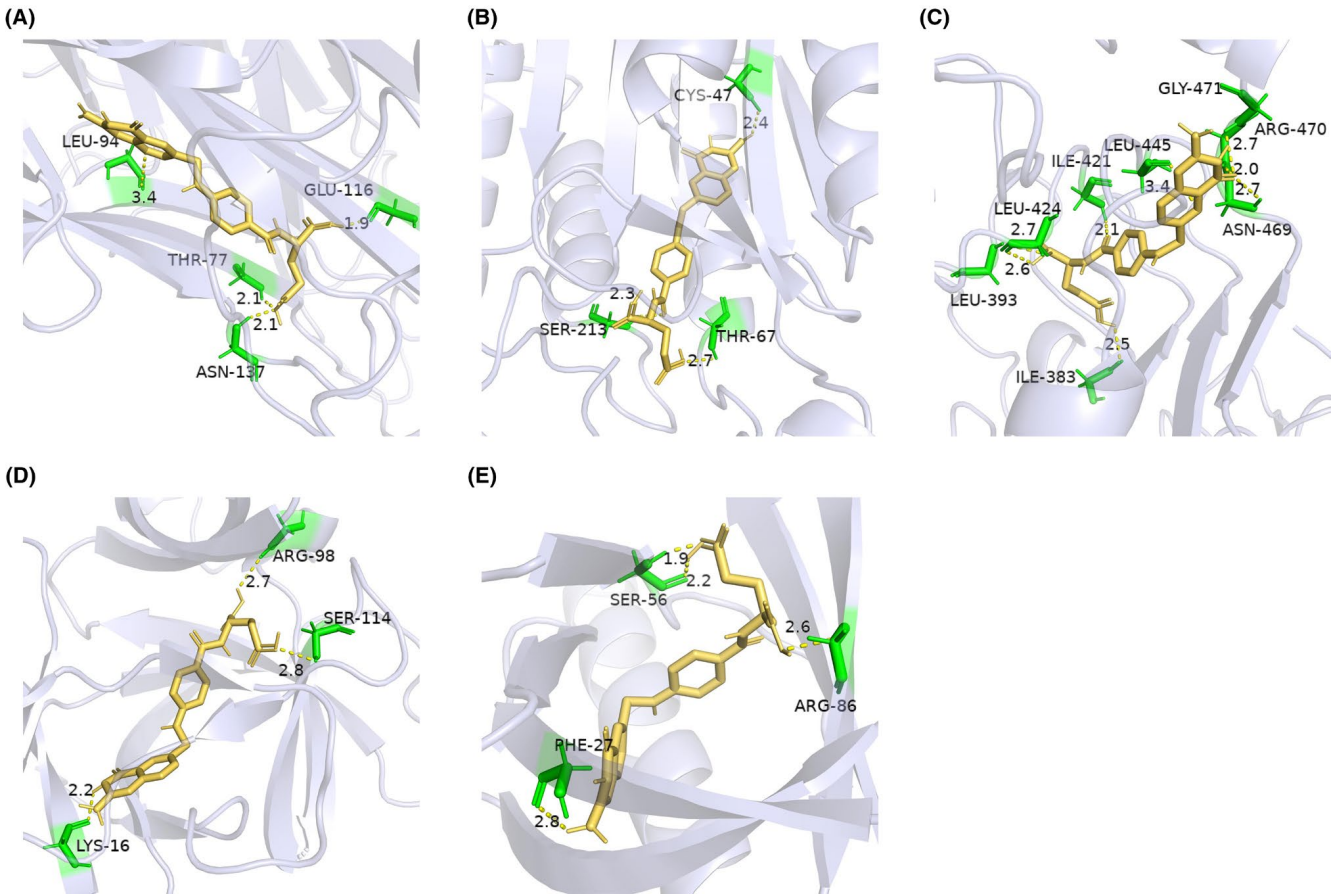
Molecular docking visualization; (A) TNF‐α and folate; (B) CASP3 and folate; (C) EGF and folate; (D) IL‐1β and folate; (E) AKT1 and folate.

Molecular docking studies were performed between folic acid and selected target proteins: TNF‐α, CASP3, EGF, IL1β, and AKT1 (Figure 8). The interactions between these targets and folic acid were systematically analyzed. The structures of the target proteins were retrieved from the RCSB PDB. The 3D conformation of folate was obtained from the PubChem database. Semi‐flexible molecular docking was conducted using AutoDockTools‐1.5.6 software. Lower molecular docking binding energies indicate stronger interactions between the drug and its target protein. Typically, a binding energy < −5 kcal/mol indicates stable docking and conformation. The results showed folate bound to all five targets with binding energies below −5 kcal/mol (Table 1), indicating effective interactions. Notably, CASP3 and TNF‐α exhibited the strongest binding affinities with folate, both at −8.5 kcal/mol. CASP3 and TNF‐α were identified as potential key targets for folate in SCI treatment and were selected for further investigation using molecular dynamics simulations.

**TABLE 1:** Docking and binding ability of folate to core targets.

	TNF‐α	CASP3	EGF	IL‐1β	AKT1
UniProt_ID	P01375	P42574	P01133	P01584	P31749
Protein_Name	Tumor necrosis factor‐α	Caspase‐3	Epidermal growth factor	Interleukin‐1 beta	Protein kinase B
PDB ID	1A8M	1CP3	1IVO	1HIB	1H10
Binding energy (kcal/mol)	−8.5	−8.5	−7.8	−7.7	−6.4

### 2.7 Clinical Trial Validation Results

**FIGURE 9:**
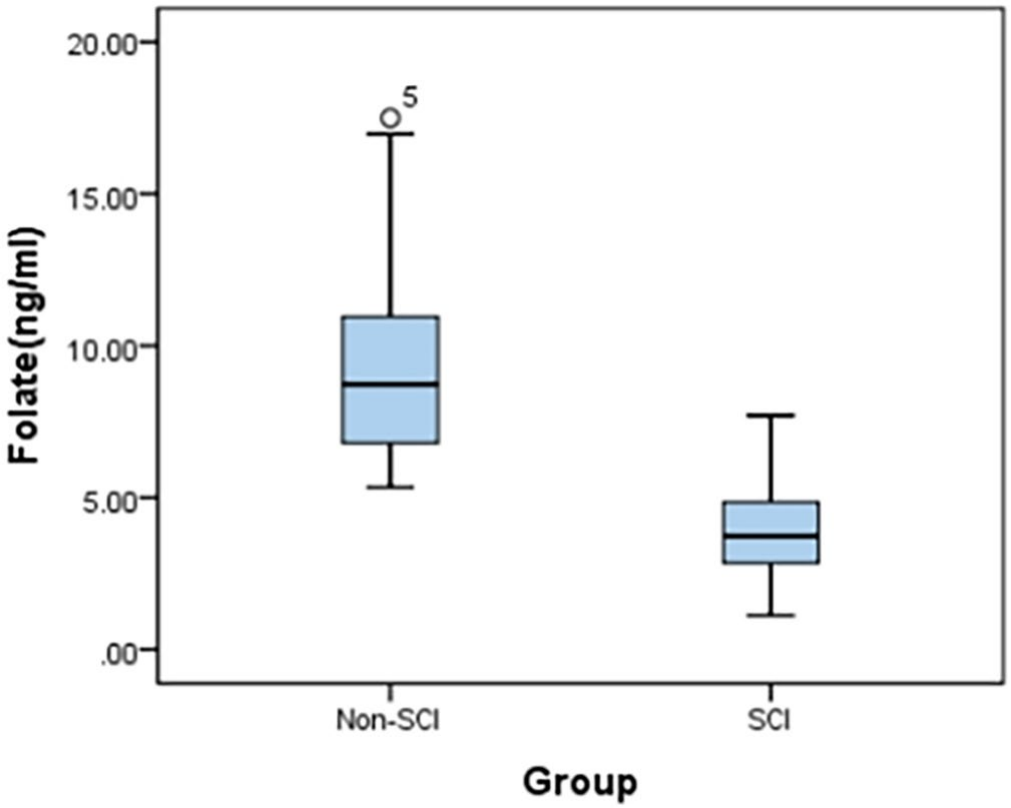
Comparative box plots of folate levels in both groups.

A cohort of 50 SCI patients (30 male, 20 female; mean age 45.82 ± 8.40 years) and 50 non‐SCI controls (28 male, 22 female; mean age 47.40 ± 7.68 years) were enrolled. No significant intergroup differences were observed in age or sex distribution (*p* > 0.05). Serum folate analysis revealed significantly lower levels in SCI patients versus controls (3.87 ± 1.60 ng/mL vs. 9.38 ± 3.03 ng/mL; *p* < 0.001; Table 2; Figure 9). Using WHO folate deficiency criteria (< 4.00 ng/mL) [18, 19], the SCI cohort's mean value fell below this threshold, with 81.0% (21/26) of cervical SCI patients exhibiting deficiency. Further analysis revealed that the cervical cord injury cohort exhibited the lowest folate levels (3.12 ± 1.24 ng/mL) among all injury segments (Cervical, Thoracic, Lumbar) (Table 3), with a significant inverse relationship between folate concentrations and lesion level (*r* = −0.58, *p* < 0.001), indicating progressively lower concentrations at more rostral levels.

**TABLE 2:** Comparative analysis of folic acid levels between the two groups (ng/mL).

Group	Sample (*n*)	Mean ± SD	Median	95% CI
Non‐SCI	50	9.38 ± 3.03	8.73	8.52–10.23
SCI	50	3.87 ± 1.60	3.73	3.82–4.98

**TABLE 3:** Folate analysis across SCI segments (Cervical, Thoracic, Lumbar).

	Sample (*n*)	Folate (ng/mL)
Cervical	26	3.13 ± 1.11
Thoracic	19	4.51 ± 1.80
Lumbar	5	5.31 ± 1.10

## 3 Discussion

SCI is characterized by high morbidity and mortality, with limited current therapeutic options. Primary pharmacological interventions include corticosteroids such as methylprednisolone; however, their clinical benefits, optimal dosing regimens, and significant adverse effects remain controversial [20]. Consequently, identifying novel and effective treatments is critically important. Folate demonstrates substantial therapeutic potential for SCI; nevertheless, its specific molecular targets and mechanisms of action remain incompletely characterized. Network pharmacology and bioinformatics approaches facilitate drug discovery, screening, and clinical translation. This study employed these methodologies to predict folate's potential therapeutic targets and mechanisms in SCI. Molecular docking further elucidated folate's interactions with these targets and its putative efficacy, providing novel perspectives and a theoretical foundation for advancing folate‐based SCI therapies.

The present study provides the first clinical evidence demonstrating that serum folate levels in the SCI group (3.87 ± 1.60 ng/mL) were significantly lower than those in the control group (9.38 ± 3.03 ng/mL) (*p* < 0.001), with the SCI group mean falling below the reference range (< 4.00 ng/mL). These findings suggest that folate deficiency represents a pathophysiological feature associated with SCI. This phenomenon likely stems from SCI‐induced disruption of sympathetic pathways, resulting in vagal predominance that triggers gastrointestinal dysmotility (including delayed gastric emptying and weakened intestinal peristalsis) [21, 22]. Consequently, this reduces secretion of gastric acid and intrinsic factor, thereby impairing folate absorption. A strong inverse correlation was observed between folate levels and injury level (*r* = −0.58, *p* < 0.001), with the lowest concentrations detected in the cervical SCI group. This may involve disruption of the Blood‐spinal cord barrier (BSCB) combined with elevated cytokine‐mediated vascular permeability at cervical segments post‐injury, potentially facilitating folate extravasation into damaged neural tissue [23].

Network pharmacology analysis identified 105 common targets between folate and SCI. Core targets (TNF‐α, IL1β, TP53, IL6, AKT1, CASP3, EGF, BCL2) and key signaling pathways (AGE‐RAGE, PI3K‐Akt, MAPK) were subsequently delineated using Cytoscape 3.9.1, suggesting folate's therapeutic roles in modulating neuroinflammation, cell survival/apoptosis, and repair signaling. TNF‐α and IL6 are pivotal mediators of inflammatory responses, while BCL2 and CASP3 critically regulate the balance between cell survival and apoptosis. GO enrichment analysis indicated that folate‐regulated target genes localize to the synaptic membrane, vesicle lumen, and endoplasmic reticulum lumen, implying that folate may enhance synaptic function and neuronal signaling through modulation of neurotransmitter synthesis, storage, and release. MF enrichment in “growth factor receptor binding”, “protein kinase regulatory activity”, and “ubiquitin ligase activity” suggests folate influences neural repair and regeneration by regulating growth factor signaling pathways and protein homeostasis. These results collectively elucidate potential mechanisms of folate in SCI repair, including attenuation of neuroinflammation, improvement of synaptic function, reduction of oxidative stress, and promotion of neuronal survival and repair. Specifically, folate may activate the PI3K/Akt signaling pathway, downregulate TNF‐α/IL1β expression, inhibit microglial M1 polarization, disrupt pro‐inflammatory cytokine feedback loops, and maintain BSCB integrity. This finding implies that targeting the AGE‐RAGE signaling pathway may represent a key anti‐inflammatory mechanism for folate in SCI repair. The AGE‐RAGE, PI3K‐Akt, and MAPK signaling pathways are all implicated in BSCB disruption and glial scar formation. Folic acid may repair the injured microenvironment through synergistic modulation of these pathways. Key mechanisms include: Activation of the AGE‐RAGE axis disrupts cellular redox homeostasis, exacerbating oxidative stress and inflammatory responses while regulating multiple cell death modalities [24, 25]. Following SCI, the PI3K‐Akt pathway critically modulates the apoptosis‐regeneration equilibrium in neurons. Folic acid potentially upregulates EGF expression, thereby enhancing AKT1 activity via an EGFR‐PI3K positive feedback loop to drive mTOR‐dependent axonal regeneration [26, 27]. The MAPK signaling pathway regulates various cellular processes such as proliferation, differentiation, cell death, survival, and inflammatory responses [28, 29]. MAPK can be activated by diverse extracellular molecules, leading to the activation of transcription factors including nuclear transcription factor‐kappa B (NF‐κB), which coordinates the induction of numerous inflammatory cytokines.

Molecular docking demonstrated binding energies below −5 kcal/mol for folate interactions with TNF‐α, CASP3, EGF, IL1β, and AKT1, indicating strong binding affinity. Notably, CASP3 and TNF‐α exhibited the highest affinity for folate (−8.5 kcal/mol). As an executioner protease, CASP3 is activated via the mitochondrial pathway during secondary SCI injury, contributing to irreversible neuronal death [30]. High‐affinity binding of folate to CASP3 may inhibit its enzymatic activity by occupying the active site, thereby blocking apoptotic cascades [31], suggesting folate may exert neuroprotective effects through direct modulation of apoptotic pathways. CASP3 also participates in inflammatory cascades; folate may consequently reduce the release of pro‐inflammatory cytokines (e.g., TNF‐α, IL1β) by attenuating its activity [32]. TNF is a core mediator of pro‐inflammatory microenvironments post‐SCI; its overexpression activates NF‐κB signaling and induces BSCB disruption [33]. Folate's high‐affinity binding to TNF may interfere with TNFR1 receptor interaction, thereby reducing cytokine release and suppressing downstream inflammatory signaling. IL1β synergistically amplifies inflammation with TNF; dual inhibition by folate may alleviate neuroinflammation by blocking inflammasome activation. As a core component of the PI3K/AKT/mTOR pathway, AKT1 activation inhibits apoptosis and promotes axonal regeneration [34]. Folate binding to AKT1 may enhance its phosphorylation through allosteric effects, facilitating regulation of downstream targets such as GSK‐3β [35]. Collectively, these findings indicate that folate promotes functional recovery after SCI through dual antioxidative stress and anti‐inflammatory mechanisms.

Previous studies have described the anti‐inflammatory effects of folate, although the underlying mechanisms remain incompletely elucidated [36]. Beneficial effects of combined micronutrient supplementation, including folate and vitamin B12, on reducing inflammation during pregnancy have been reported, acting at the level of inflammatory cytokines [37]. Kolb et al. demonstrated that folate deficiency enhances pro‐inflammatory cytokine output in RAW264.7 monocytes, suggesting that deficiency may amplify pro‐inflammatory signaling within the monocyte–macrophage lineage, potentially exacerbating cardiovascular disease [38]. Folate is proposed to indirectly suppress inflammatory responses by modulating intracellular signaling pathways. For instance, studies indicate that folate attenuates LPS‐induced inflammation in microglia by blocking NF‐κB and JNK activation, and by upregulating IL‐10‐dependent suppressors of cytokine signaling (SOCS) protein expression via the p38 MAPK pathway, thereby inhibiting the release of pro‐inflammatory mediators [39]. However, this study provides the first evidence suggesting that folate may also exert neuroprotective effects through the direct binding and modulation of core inflammatory and apoptotic target proteins. The direct binding of folate to TNF may inhibit the formation of its functional trimer or occupy the TNFR1 interaction interface, consequently suppressing TNF‐mediated activation of the NF‐κB pathway. This finding aligns closely with prior reports of folate inhibiting NF‐κB but reveals a more upstream molecular target. Furthermore, folate binding to CASP3 likely inhibits the activity of this key apoptotic executioner protein, potentially by occupying its catalytic domain (e.g., at the Cys163 residue), thereby blocking the neuronal apoptotic cascade.

This study first systematically evaluated the mechanism of folate in SCI treatment by integrating retrospective clinical research and network pharmacology. Findings revealed significantly lower serum folate levels in SCI patients compared to the non‐SCI group (*p* < 0.001). A strong inverse correlation was observed between folate levels and injury level (*r* = −0.58, *p* < 0.001). Network pharmacology analysis indicated that folate may inhibit apoptosis‐executing protein activity and reduce pro‐inflammatory factor release through high‐affinity binding to core targets including CASP3 (binding energy: −7.2 kcal/mol) and TNF‐α (−6.8 kcal/mol). This suggests folate deficiency may compromise anti‐apoptotic/anti‐inflammatory capacities, accelerating neuronal death in secondary injury. TNF‐α and IL‐1β drive BSCB disruption during acute SCI [40], while molecular docking demonstrated high folate affinity for TNF‐α/IL1β, implying analogous pharmacological activity.

Collectively, this evidence supports that folate supplementation may synergistically improve the SCI microenvironment via a “multi‐target/multi‐pathway” paradigm. Based on these mechanisms, folate supplementation is recommended for SCI patients.

Good nutrition may be crucial for patients with SCI; however, we lack data demonstrating the efficacy of such recovery. Future clinical trials are warranted to clarify the dose–response relationship and long‐term neurological outcomes, while folate‐deficient SCI animal models could further validate the mechanisms underlying folate treatment for SCI.

## 4 Materials and Methods

### 4.1 Acquisition of Active Ingredients and Targets of Folate

The compound name “folate” was entered into the TCMSP database (https://tcmspw.com/tcmsp.php) [41] with screening criteria set to oral bioavailability ≥ 30% and drug‐likeness ≥ 0.18 to retrieve its active ingredients and corresponding targets [42]. The SwissTargetPrediction (http://www.swisstargetprediction.ch/) [43], ChEMBL (https://www.ebi.ac.uk/chembl/) [44], and PubChem (http://pubchem.ncbi.nlm.nih.gov/) [45] databases were utilized to supplement active ingredients not included in TCMSP. Potential targets for these ingredients were predicted using the SwissTargetPrediction online platform [46] (probability > 0.1).

### 4.2 Acquisition of SCI‐Related Targets

Two disease databases, GeneCards (http://www.genecards.org/) [47] and OMIM (http://omim.org/) [48], were employed to search for disease‐associated genes using the keyword “Spinal cord injury”. Target genes with a relevance score > 1 were included. The obtained disease‐related targets were deduplicated and integrated, yielding a total of 548 unique targets.

### 4.3 Construction of the “Drug‐Ingredient‐Target” Network and Screening of Active Ingredients

The intersection between folate targets and SCI‐related genes was identified using the VennDiagram package within Bioconductor in R software, and a Venn diagram was constructed to visualize folate's potential therapeutic targets for SCI [49]. Module analysis of the intersecting genes was performed using the CytoNCA plugin in Cytoscape 3.9.1 to screen for core active ingredients. The drug‐target interaction network was analyzed to identify folate's primary active components.

### 4.4 Construction of the PPI Network and Identification of Core Genes

The potential therapeutic targets of folate for SCI were input into the STRING database (https://stringdb.org/) [50] with the organism specified as *Homo sapiens* . A protein–protein interaction (PPI) network was constructed using a maximum confidence threshold ≥ 0.4 as the filtering criterion, generating a visual representation of the interactions among target proteins [51]. Using data exported from STRING, core targets were identified in Cytoscape 3.9.1 by evaluating degree, closeness centrality, and betweenness centrality metrics. The highest‐scoring target was selected as the key target for subsequent studies [52].

### 4.5 GO Function and KEGG Pathway Enrichment Analysis

Gene Ontology (GO) annotation and Kyoto Encyclopedia of Genes and Genomes (KEGG) pathway enrichment analyses were conducted using the DAVID database (https://david.ncifcrf.gov/) [53]. GO analysis encompasses biological processes (BP), cellular components (CC), and molecular functions (MF). Terms with a *p*‐value < 0.05 were considered significant [54]. The top 10 enriched GO terms and the top 30 enriched KEGG pathways, sorted by term, gene count, and P‐value, were selected. Results were visualized using an online platform (http://www.bioinformatics.com.cn) [55].

### 4.6 Molecular Docking Analysis

Molecular docking technology employs computational simulation to predict intermolecular interactions based on spatial and energetic complementarity. In this study, the two‐dimensional (2D) structure files of small molecule ligands were downloaded from the PubChem database (https://pubchem.ncbi.nlm.nih.gov/) [43]. These structures were optimized and converted into three‐dimensional (3D) structures using ChemBio3D 14.0.0 software. The Protein Data Bank (PDB) files of receptor proteins were downloaded from UniProt (https://www.uniprot.org/) [56] and the PDB database (http://www.rcsb.org/) [57]. Water molecules and ligands were removed from the receptor structures using PyMOL 2.4 (https://pymol.org/2/) [57]. Hydrogen atoms and Gasteiger charges were added to both ligands and receptors using AutoDockTools 1.5.6 software (http://autodock.scripps.edu/) [58]. Docking calculations were performed using the AutoDock Vina program (http://vina.scripps.edu/) [59]. The binding affinity and activity between targets and active compounds were evaluated based on the docking scores; a binding energy less than −5 kcal/mol was considered indicative of feasible binding. The molecular docking results were visualized using PyMOL software.

### 4.7 Clinical Trial Validation

This study was an observational study, not a prospectively designed study. Patient serum folate level measurement data were derived from routine clinical testing performed in the Hematology Department of Tianjin Hospital.

*Detailed methodological description*: Serum folate concentrations were measured using Chemiluminescent Immunoassay (CLIA). The assay was performed using the Roche Cobas e 411 fully automated electrochemiluminescence immunoassay analyzer and the corresponding folate detection reagent kit. The detection principle is based on a competitive immunoassay format: folate in the sample competes with a folate analog conjugated to a luminescent label (labeled antigen) in the reagent for binding sites on folate‐specific antibodies immobilized on magnetic particles (solid‐phase antibody). The higher the folate concentration in the sample, the less labeled antigen binds to the antibody; conversely, the lower the folate concentration in the sample, the more labeled antigen binds to the antibody. Specific operations strictly followed the Standard Operating Procedures provided by the instrument manufacturer and the laboratory's internal quality control protocols. Results are reported in ng/mL or nmol/L.

The research protocol was approved by the hospital's Ethics Committee (Approval No.: 2025‐004).

#### 4.7.1 Group Design (Table 4)

**TABLE 4:** Inclusion and exclusion criteria for both groups.

Group	Inclusion criteria	Exclusion criteria
SCI (*n* = 50)	1. Age 18–65 years 2. Met ASIA 2019 diagnostic criteria for traumatic SCI	1. Non‐traumatic SCI (tumor/infection/etc.) 2. Severe hepatic or renal insufficiency 3. History of folate supplementation (past 3 months)
Non‐SCI Group (*n* = 50)	1. Age 18–65 years 2. Normal hematological/biochemical parameters 3. No history of SCI	1. Active autoimmune diseases 2. Pregnancy or lactation 3. Vitamin B12 deficiency (serum B12 < 200 pg/mL), history of folate supplementation (past 3 months)

#### 4.7.2 Outcome Measures

Serum folate levels were quantitatively analyzed.

#### 4.7.3 Statistical Analysis

Using *t*‐tests (for normally distributed continuous variables) or Mann–Whitney *U*‐tests (for non‐normally distributed variables). Correlational analyses: Pearson's correlation coefficient (*r*) for normally distributed data. Spearman's rank correlation coefficient (*ρ*) for non‐normally distributed or ordinal data.

Nomenclature of Targets and Ligands.

Key protein targets and ligands in this article are hyperlinked to corresponding entries in http://www.guidetopharmacology.org, the common portal for data from the IUPHAR/BPS Guide to PHARMACOLOGY, and are permanently archived in the Concise Guide to PHARMACOLOGY 2019/20 [60].

## 5 Conclusions

This study provides key molecular mechanistic evidence for the application of folate in SCI treatment and establishes a theoretical foundation for targeted intervention strategies. The results demonstrate that folic acid exerts neuroprotective effects by coordinately modulating neuronal apoptosis, inflammatory responses, and oxidative stress pathways. Notably, clinical data indicate prevalent folic acid deficiency in SCI patients. This nutritional deficit may exacerbate secondary injury cascades, thereby impeding neurological functional recovery. Based on the above mechanisms and clinical correlations, it is recommended to incorporate folic acid supplementation into comprehensive SCI therapeutic management.

## Author Contributions

Conceptualization: Xiaolei Chu and Jiajia Liang. Methodology: Jiaojiao Sun and Zheng Xing. Software: Wenjie Liu and Lei Zhang. Validation: Qingwen Li and Jiajia Liang. Resources: Qi Li. Data curation: Zheng Xing. Writing – original draft preparation: Xiaolei Chu. Visualization: Jiajia Liang. Funding acquisition: Qi Li and Qingwen Li. All authors have read and agreed to the published version of the manuscript.

## Funding

Biology and Information Convergence—National Key R&D Program of China（(2023YFF1205200), Project participant: Zheng Xing. Natural Science Foundation of Tianjin City (22JCYBJC00210), Project leader: Qi Li. Natural Science Foundation of Tianjin City (22JCYBJC00220), Project leader: Xiaolei Chu.

## Ethics Statement

The study was conducted in accordance with the Declaration of Helsinki, and approved by the Ethics Committee of Tianjin Hospital, Tianjin, China (Medical Ethics Review 2025‐004).

## Consent

Informed consent was obtained from all subjects involved in the study.

## Conflicts of Interest

The authors declare no conflicts of interest.

## Data Availability

The original contributions presented in the study are included in the article/Supporting Information; further inquiries can be directed to the corresponding authors.
